# Assessment Strategies to Evaluate the Mediterranean Lifestyle: A Systematic Review

**DOI:** 10.3390/nu14194179

**Published:** 2022-10-08

**Authors:** Elisabet Montero-Sandiego, Rosario Ferrer-Cascales, Nicolás Ruiz-Robledillo, Borja Costa-López, Cristian Alcocer-Bruno, Natalia Albaladejo-Blázquez

**Affiliations:** Department of Health Psychology, Faculty of Health Science, University of Alicante, 03690 Alicante, Spain

**Keywords:** Mediterranean Lifestyle, evaluation, questionnaire, index

## Abstract

The Mediterranean Lifestyle (MLS) has been related to better health and quality of life. However, there is no consensus on how to assess this lifestyle. The main objective of this work was to systematically review the methodology used in different studies on the evaluation of the MLS. The specific objectives were (1) to analyze the MLS components evaluated in previous studies, (2) to explore the assessment instruments available for the analysis of the MLS, and (3) to identify the psychometric properties of these instruments. The search was carried out using the PubMed, Scopus, Web of Science, and ScienceDirect databases with the purpose of identifying those published articles in which the MLS was assessed. The review included 26 studies linked to the assessment of the MLS. Of these studies, only four exclusively used a tool to analyze MLS components globally. These studies included two questionnaires and three different indexes. None of them, however, evaluated all of the recognized MLS components, and food preparation was the least frequently evaluated component. Given the clear importance of analyzing MLS adherence and the lack of consensus in previous research, an evaluation tool needs to be created to comprehensively assess all of the MLS dimensions by means of appropriate psychometric properties.

## 1. Introduction

Lifestyle (LS) is considered to be a set of repeated behavioral patterns maintained over time that characterize an individual’s way of life [1]. The term has gained considerable relevance over recent years due to its close relationship with distinct pathologies and the higher levels of morbidity and mortality [2,3]. Lifestyle factors, such as physical activity, alcohol consumption, smoking, sleep, and diet have been found to be predictors of health status in both the general and the clinical population [4]. However, few studies have performed comprehensive analyses of the different so-called healthy lifestyles, such as the Mediterranean Lifestyle (MLS) [5]. This lifestyle, denominated after the countries situated near the Mediterranean Sea [2], displays the following factors: a high adherence to the Mediterranean Diet (MD); regular physical activity; good hydration and adequate rest (including regular naps); the consumption of seasonal and locally grown products; participation in food preparation and culinary activities; and frequent social interactions [2]. This lifestyle is the result of the interactions between these factors. Not only does the Mediterranean Diet pyramid incorporate recommendations regarding the frequency people should consume certain foods, and the amount of those foods, but it also refers to other dimensions that comprise the traditional Mediterranean life [6]. The MLS extends beyond a simple dietary pattern and is based on the interactions of different aspects of a healthy lifestyle. Different parameters, such as socialization, physical activity, leisure activities, appropriate rest, and, of course, diet, interact to create a healthy lifestyle, as in the case of an MLS [4]. In this way, different studies, although in isolation, have shown how the various components of the MLS are associated with better health and quality of life. For example, recent research has shown how meals shared with family members are associated with healthy weight and better eating habits [7]. Likewise, culinary activities constitute a behavior of great importance for health promotion, especially among children, as there are studies that show that it favors increased vegetable consumption [2]. In turn, the use of seasonal and locally grown products, another component of the MLS, allows avoiding the consumption of foods that have been processed for their maintenance during transportation and storage. Such processing negatively affects the nutrient content of the food, unlike local products that, after harvesting, are sold in a short time, preserving the freshness, taste, and quality of the product [2].

In relation to the practice of physical activity, there are very many studies that have shown how it is one of the most important protective factors against the development of numerous pathologies, reducing the risk of mortality and increasing life expectancy [8,9]. On the other hand, adequate night rest and characteristic naps are also protective factors against mortality from cardiovascular diseases, considering that both excess as well as lack of rest hours can become detrimental to health [5]. Likewise, socialization and participation in collective activities also provide the same benefits among the population, producing an increase in quality of life [2]. It can therefore be said that the MLS is a complex lifestyle formed by the interaction of different factors that provide great health benefits. Such benefits generally have been attributed only to dietary aspects, in which their protective role against diseases, such as diabetes, cancer, cardiovascular pathologies, obesity, and depression, is confirmed, without taking into account the other factors that configure the MLS [1,7]. Therefore, the MLS can be conceptualized as a complex lifestyle that is based on the interaction of different factors, which provide major combined health benefits. In fact, the MLS is considered a protective factor that reduces the risk of mortality and increases life expectancy [8,9].

According to numerous studies, the benefits of adhering to the MLS include a significant improvement in quality of life [10]. In this regard, many studies have shown that adherence to the MLS acts as a protective factor against chronic non-communicable diseases, including cardiovascular diseases, type 2 diabetes, hypertension [11,12,13,14,15], and metabolic syndrome [16], showing a lower prevalence of these diseases among people with strong adherence to the MLS [16]. In fact, the study by Hershey et al. showed that greater adherence to the MLS can be associated with 41% less mortality from cardiovascular disease [11]. Hence, MLS adherence acts as a protective factor against chronic diseases, especially in the elderly [10], given that adherence to this lifestyle is significantly associated with a lower risk of cardiovascular disease mortality [11,12,13,14,15]. Moreover, as previously indicated, adherence to the MLS is also closely related to a lower prevalence of metabolic syndrome [17] and has been linked to a lower development of glucose disorders in pregnant patients, subsequently reducing the rate of gestational diabetes [18]. In this sense, MLS adherence reduces the development of postpartum glucose disorders by 25%, and specifically by 35% in the rate of development of type 2 diabetes mellitus among women who have had gestational diabetes [18].

As for clinical populations, adherence to the MLS has been associated with an improved health status in renal disease groups that are not dialysis-dependent [16]. Adherence to the MLS among non-dialysis-dependent renal patients prevents progression of the disease [16]. Moreover, in a series of studies, Georgoulis et al. stated that adherence to the MLS is related to an improved cardiometabolic profile in patients with severe obstructive sleep apnea [19,20,21]. In this sense, a higher adherence to this lifestyle has been related to a reduction in the apnea and hypopnea index in daytime symptomatology, and it has been associated with an improvement in quality of life [19,20,21].

It should be added that adherence to the MLS not only has physical benefits, but also mental benefits, as it has been shown that adherence to the MLS reduces the risk of depression by 50% [22].

Despite the recognized importance of evaluating adherence to the MLS in different populations given its health benefits, to the best of our knowledge, there is no consensus as to the best strategy to reliably assess it. While some prior studies have used different questionnaires to evaluate the components of the MLS [10,19,20,21,23,24,25,26,27,28], others have used a specific index or questionnaire to assess global adherence to the MLS. However, little information exists with regard to the psychometric properties of the questionnaires created to examine the MLS [5]. Moreover, not all dimensions of the MLS have been included in the previously used evaluation strategies.

Given that past studies have relied on numerous heterogeneous assessment strategies and consensus has not yet been reached regarding the most valid and reliable strategy for the analysis of this type of lifestyle, this study attempted to systematically review and analyze the methodologies that were used in different studies to evaluate the MLS for both non-clinical and clinical populations. Moreover, the study was aimed to determine which components of the MLS were most frequently evaluated in past studies and which assessment instruments are currently being used to analyze these MLS components. Finally, the study also attempted to identify the psychometric properties of the available instruments that globally assess the MLS.

## 2. Materials and Methods

This study used a systematic review methodology that was based on the PRISMA statement [29].

### 2.1. Search Strategy

The main objective of the search strategy was to detect the published studies available in full text. The first step consisted of electronic searches carried out between December 2021 and May 2022 in the following databases: PubMed, Web of Science, Scopus, and Science Direct. This search strategy was designed to obtain original studies published on the assessment methods for MLS. A bulk search strategy was used by applying both descriptors and keywords in the titles and abstracts. Additionally, no date restrictions were applied to the articles’ year of publication. Table 1 shows the search strategies used in the different databases.

### 2.2. Inclusion and Exclusion Criteria

Inclusion criteria were (I) original articles in which at least two of the following MLS components were evaluated following the criteria proposed by Diolintzi et al. [2]: adherence to the Mediterranean Diet (MD), consumption of seasonal and locally grown products, participation in food preparation and culinary activities, regular physical activity, good hydration and adequate rest, including regular naps, and socialization; (II) articles that were available in full text and written in English or Spanish.

Exclusion criteria were (I) articles that were not related to the subject of the study; (II) articles having unreported results; (III) articles that did not refer to MLS assessment; (IV) articles that only referred to the Mediterranean Diet (MD); (V) articles that were reviews and meta-analyses; (VI) documents that were doctoral theses, reports, or conference summaries; and (VII) books and book chapters.

### 2.3. Selection of Studies

Once the search was performed in the databases, duplicates were discarded, as well as all works presented at congresses, reports, doctoral theses, and book chapters, among others.

The abstracts that were identified through the bibliographic search were independently evaluated by two authors to confirm whether or not the articles were valid according to the review’s inclusion and exclusion criteria. Two authors of this paper evaluated each article independently and discrepancies were resolved by consulting with a third author.

### 2.4. Assessing the Methodological Quality of the Studies Included in the Review

The quality of each study was analyzed by applying a commonly used battery of instruments for the evaluation of methodological quality, CADIMA, which was developed at the Joanna Briggs Institute of the University of Adelaide, Australia [30]. This battery provides different evaluation instruments for each type of study design. Specifically, the instrument used for cross-sectional studies includes eight items, the instrument used for cohort studies has eleven items, and the instrument used for randomized trials contains thirteen items. All instruments include a response scale with four response options: “yes”, “no”, “unclear”, or “not applicable”. Two authors of this paper independently assessed the quality of each study included in the review and discrepancies were resolved by consulting with a third author. The results reflected an inter-subject reliability of between 0.60 and 0.85, according to Cohen’s Kappa statistical parameter.

Tables S1–S3 in the Supplementary Material reveal the methodological quality of the assessment for the cross-sectional studies: randomized and cohort trials that were included in this review.

### 2.5. Data Extraction

The sample information extracted from each article consisted of the following elements: sample size, gender, origin, and age of the population under study. Regarding the information on the assessment methods, we extracted the Mediterranean Lifestyle components, the evaluation strategies used to analyze the MLS, the MLS evaluation instruments applied, and their psychometric properties. We also considered the study type and objective.

## 3. Results

### 3.1. Search Results

Table 2 shows the search strategies used in the different databases and details the corresponding number of total articles extracted per database.

**Figure 1:**
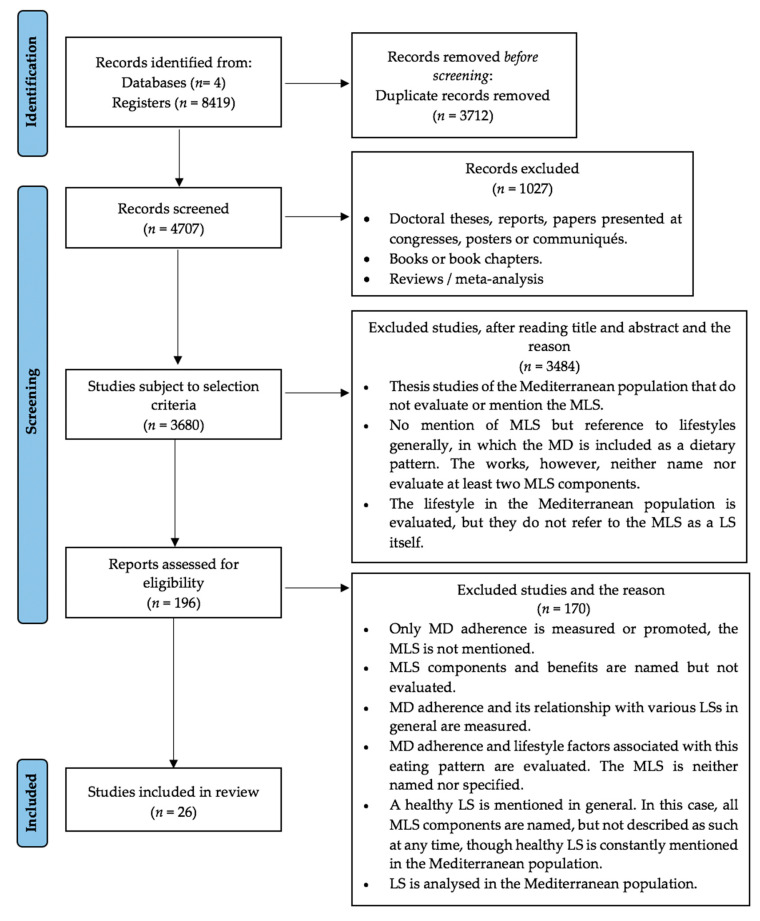
PRISMA flow diagram of studies evaluated in the systematic review.

As can be seen in Figure 1, a total of 8419 articles were obtained. After discarding duplicates, the total number was reduced to 4707 articles. A total of 3680 articles remained after discarding doctoral theses, reports, book chapters, etc. Following a review of the titles and abstracts, an additional 3484 articles were excluded. These articles were discarded mainly because they did not mention the MLS. Instead, they referred generally to lifestyles and included MD as a dietary pattern but did not name nor evaluate a minimum of two MLS components. In many other articles, the main objective was to evaluate the Mediterranean population’s lifestyle, but without referencing the MLS as an LS. In these studies, the MD was the only characteristic relating directly to the Mediterranean population’s LS, without considering any other characteristic component of the MLS.

After reading the full texts of the articles, an additional 170 were excluded. The reasons for their exclusion were as follows:

- MD adherence and its relationship with various LSs, in general, were measured. However, the MLS was not mentioned.
- MLS components and benefits were named but not evaluated.
- The Mediterranean population’s LS was measured, and the MD was included, but no reference was made to the MLS nor its components.
- A healthy LS was mentioned in general. In this case, all MLS components were named but were not described as such at any time. Instead, a healthy LS in the Mediterranean population was constantly referred to.

Finally, after a rigorous search, a total of 26 articles were included in the review.

### 3.2. Characteristics of the Studies Included in the Review

Table 3 lists all of the studies that were used in the final review. The following information is listed per study: authors, study design, year of publication, country, population type, sample size, gender, age, and study objective.

Most of the articles considered a general population living in the Mediterranean Basin. However, some studies included participants having a specific pathology, such as: dementia [23], Chronic Renal Insufficiency [16], obesity and metabolic syndrome or sleep apnea [10,19,20,21], gestational diabetes [18], type 2 diabetes mellitus [31,32], and fatty liver disease [27].

The article having the largest number of participants had a sample size of 174,209 individuals [28]. In contrast, the study with the smallest sample size had a total of 63 participants [27]. With the exception of two articles, all of the articles specified the gender of the participants.

Most of the articles focused on a middle-aged population, except for one article, which focused on students aged 6 to 18 years old [28]. The study with the largest age range covered <30 to >70 years [33]. Finally, one article did not specify the age of its participants [26].

The main objective of all the articles was to evaluate the MLS in order to analyze the relationship between adherence to this lifestyle and other variables, either with other lifestyles [23,24,33], with the symptomatology of certain pathologies [10,11,12,13,14,15,16,17,22,25,28], or with the effectiveness of an intervention program in which the MLS was implemented in the daily lives of participants, comparing certain variables before and after the implementation of this LS [18,19,20,21,27,31,32]. Another study focused on the assessment of changes in adherence to the MLS after the COVID-19 pandemic [34]. Two other studies considered the designing of a questionnaire to measure MLS adherence and the verification of its reliability [5,35].

Of the studies included in this review, Table 3 reveals that nine were cross-sectional [12,16,17,23,24,25,28], ten were randomized controlled trials [10,15,18,19,20,21,27,31,32,35], and the remaining seven were cohort studies [5,11,13,14,22,26,33].

### 3.3. Conceptual Suitability

As illustrated in Table 3, the majority of the countries considered in the studies were part of the Mediterranean Basin—e.g., Spain, Italy, Greece, and Croatia—with the exception of the US and Australia, where a total of four studies were conducted [12,16,31,32]. In two other articles, collaboration took place between Mediterranean and non-Mediterranean countries, specifically Spain and Greece and the US [17,28].

The purpose of all of the studies was to evaluate the population’s MLS adherence in order to promote this LS, and thereby use the results to demonstrate that high MLS adherence benefits the population’s health, acting as a protective factor against various diseases, especially at a cardiovascular level. Therefore, the aim of the articles was both to promote MLS adherence among the population and to demonstrate its protective effect against different pathologies.

### 3.4. Applicability

In the study by Katsagoni et al. [28], the population sample was composed of students aged 16 to 18 years. Online questionnaires were used in the students’ classrooms and there were trained teachers and/or Information Technology instructors who had been previously trained on the specific guidelines in order to help students correctly complete the questionnaires.

The remainder of the study questionnaires were administered by a trained dietitian, trained personnel, or through online questionnaires, in which the participants responded without the need for any external assistance.

**Table 3:** General characteristics of the studies included in the review.

Author(s)	Year	Country	Type of Population	Sample Size	Gender	Age				Objective of the Study	Study Design
						Average	SD	Range	Median		
Anastasiou et al. [23]	2018	Greece	Elderly population with dementia	*n* = 1716	Men: *n* = 693 Women: *n* = 1023	72.9	6.1	-	-	To investigate the relationship between cognitive function and LS, based on the MLS.	Cross-sectional study
Baldini et al. [24]	2009	Italy	University students from two Mediterranean regions (Italy/Spain)	*n* = 210	Men: *n* = 85 Women: *n* = 125	-	-	22–32	-	To compare the MLS between young Spaniards and Italians in order to check which group has the best LS.	Cross-sectional study
Bonaccio et al. [34]	2022	Italy	Elderly population	*n* =4400	Men: *n* = 1863 Women: *n* = 2537	-	-	65–99	-	To evaluate dietary changes during the COVID-19 pandemic.	Cross-sectional study
Bouzas et al. [10]	2020	Spain	Patients with obesity and metabolic syndrome	*n* = 6355	Men: *n* = 3268 Women: *n* = 3087	-	-	55–75	-	To analyze the association between adherence to the MLS and weight loss.	Randomized controlled trial
Bowden et al. [16]	2021	Australia	Patients with Chronic Renal Insufficiency	*n* = 99	Men: *n* = 64 Women: *n* = 35	73.2	10.5	-	-	To assess adherence to the MLS and its association with cardiometabolic markers and renal function in individuals with chronic renal failure who are not dependent on dialysis.	Cross-sectional study
Georgousopoulou et al. [25]	2017	Greece	Elderly population in the Mediterranean Basin	*n* = 2749	Men: *n* = 1369 Women: *n* = 1380	-	-	65–100	-	To assess the cardiovascular effects of adherence to the MLS.	Cross-sectional study
Georgoulis et al. [19]	2020	Greece	Overweight/obese population + obstructive sleep apnea	*n* = 187 Standard LS *n* = 65 MD *n* = 62 MLS *n* = 60	Men: *n* = 141 Women: *n* = 46	49	10	-	-	To compare patients with severe obstructive sleep apnea by performing one or more of these three programs for six months: follow-up of a standard LS, MD adherence, or MLS adherence (MIMOSA Study).	Randomized controlled trial
Georgoulis et al. [20]	2020	Greece	Overweight/obese population + obstructive sleep apnea	*n* = 187 Standard LS *n* = 65 MD *n* = 62 MLS *n* = 60	Men: *n* = 141 Women: *n* = 46	49	10	-	-	To measure the efficacy of interventions in patients with severe obstructive sleep apnea by implementing an MD or MLS adherence program (MIMOSA Study).	Randomized controlled trial
Georgoulis et al. [21]	2021	Greece	Overweight/obese population + obstructive sleep apnea	*n* = 187 Standard LS *n* = 65 MD *n* = 62 MLS *n* = 60	Men: *n* = 141 Women: *n* = 46	49	10	-	-	To assess the efficacy of the MIMOSA program through MD or MLS adherence, and the prescription of continuous positive airway pressure (CPAP).	Randomized controlled trial
Grosso et al. [26]	2017	Italy	General population	Proposal of 1500 participants	-	-	-	-	-	To provide data to increase knowledge about the prevalence, incidence, and risk factors of age-related disorders in the Mediterranean region.	Cohort study
Hershey et al. [11]	2020	Spain	Graduate students	*n* = 20,494 Divided into four groups, according to the degree of MLS adherence, from lowest to highest adhesion: Q1: *n* = 6390 Q2: *n* = 5783 Q3: *n* = 4820 Q4: *n* = 3501	Men: *n* = 8008 Women: *n* = 12,486 Q1: Men: *n* = 2681 Women: *n* = 3709 Q2: Men: *n* = 2348 Women: *n* = 3435 Q3: Men: *n* = 1857 Women: *n* = 2963 Q4: Men: *n* = 1122 Women *n* = 2379	Q1: 37.25 Q2: 37.79 Q3: 37.94 Q4: 37.76	Q1: 12.46 Q2: 12.34 Q3: 12.34 Q4: 12.39	-	-	To associate the relationship between MLS and the causes of mortality.	Cohort study
Hershey et al. [17]	2021	US	US firefighters	*n* = 249 Divided into three groups according to MLS adherence, from lowest to highest adherence: T1: *n* = 90 T2: *n* = 99 T3: *n* = 60	Men: *n* = 236 Women: *n* = 13 T1: Men: *n* = 88 Women: *n* = 2 T2: Men: *n* = 92 Women: *n* = 7 T3: Men: *n* = 56 Women: *n* = 4	T1: 46.92 T2: 46.66 T3: 46.56	T1: 6.98 T2: 7.57 T3: 8.08	-	-	To associate the relationship between adherence to the MLS and metabolic syndrome in a non-Mediterranean population (US firefighters).	Cross-sectional study
Hershey et al. [36]	2021	Spain/US	General population	*n* = 15,279 Q1: *n* = 4865 Q2: *n* = 4387 Q3: *n* = 3520 Q4: *n* = 2507	Men: *n* = 6100 Women: *n* = 9179 Q1: Men: *n* = 1946 Women: *n* = 2919 Q2: Men: *n* = 1750 Women: *n* = 2637 Q3: Men: *n* = 1404 Women: *n* = 2116 Q4: Men: *n* = 1000 Women: *n* = 1507	Q1: 37.0 Q2: 37.0 Q3: 37.0 Q4: 37.0	Q1: 11.8 Q2: 11.5 Q3: 11.5 Q4: 11.7	-	-	To associate the relationship between the Mediterranean Lifestyle and the risk of depression.	Cohort study
Katsagoni et al. [28]	2020	Greece	Students aged 6 to 18 years	*n* = 174,209 Divided into three groups according to MLS adherence, from lowest to highest adherence: Low: *n* = 26,488 Average: *n* = 108,229 High: *n* = 39,492	Men: *n* = 89,174 Women: *n* = 85,035 Low: Men: *n* = 13,668 Women: *n* = 12,820 Average: Men: *n* = 55,089 Women: *n* = 53,140 High: Men: *n* = 20,417 Women: *n* = 19,075	-	-	-	Low: 11.8 Average: 11.2 High: 10.9	To analyze the relationship between adherence to the MLS and obesity in children and adolescents.	Cross-sectional study
Katsagoni et al. [27]	2018	Greece	Patients with fatty liver	*n* = 63 GC: *n* = 21 MDG MD: *n* = 21 MLG: *n* = 21	Men: *n* = 43 Women: *n* = 20 GC: Men: *n* = 13 Women: *n* = 8 MDG: Men: *n* = 13 Women: *n* = 8 MLG: Men: *n* = 17 Women: *n* = 4	-	-	-	GC: 47 MDG: 44 MLG: 48	Intervention to improve the weight of patients with fatty liver.	Randomized controlled trial
Lan et al. [12]	2020	USA	Active firefighters	*n* = 92 Divided into three groups according to MLS adherence, from lowest to highest adherence: Low: *n* = 10 Medium: *n* = 55 High: *n* = 27	Men: *n* = 89 Women: *n* = 3 Low: Men: *n* = 10 Women: *n* = 0 Medium: Men: *n* = 53 Women: *n* = 2 High: Men: *n* = 26 Women: *n* = 1	Low: 31.9 Medium: 27.6 High: 29.4	Low: 8.2 Medium: 3.9 High: 5.5	-	-	To analyze the relationship between adherence to the MLS and cardiovascular disease risk factors.	Cross-sectional study
Marventano et al. [33]	2017	Italy	Patients randomly selected from the lists of a group of doctors	*n* = 1952 Divided into four groups according to MD adherence, from lowest to highest adherence: Q1: *n* = 471 Q2: *n* = 600 Q3: *n* = 606 Q4: *n* = 285	Men: *n* = 813 Women: *n* = 1139 Q1: Men: *n* = 203 Women: *n* = 208 Q2: Men: *n* = 248 Women: *n* = 352 Q3: Men: *n* = 250 Women: *n* = 365 Q4: Men: *n* = 112 Women: *n* = 163	Range <30: 11.5 Range 30–39: 11.6 Range 40–49: 12.1 Range 50–59: 12.1 Range 60–69: 12.6 Range >70: 12.1	Range <30: 2.4 Range 30–39: 2.5 Range 40–49: 2.4 Range 50–59: 2.4 Range 60–69: 1.9 Range >70: 2.4	<30 30–39 4–49 50–59 60–69 >70	-	To evaluate the level of MD adherence and PA and its determinants in the Mediterranean healthy Eating, Aging, and Lifestyle (MEAL) study.	Cohort study
Mata-Fernández et al. [13]	2021	Spain	University graduates	*n* = 18,419 Divided into three groups according to MEDLIFE score: Low: *n* = 2928 Average: *n* = 9548 High: *n* = 5943	Men: *n* = 7267 Women: *n* = 11,152 Low: Men: *n* = 1232 Women: *n* = 1696 Medium: Men: *n* = 3976 Women: *n* = 5572 High: Men: *n* = 2059 Women: *n* = 3884	Low: 36.9 Medium: 38.3 High: 38.3	Low: 11.7 Medium: 12.2 High: 12.2	-	-	To assess the relationship between adherence to the MLS and the incidence of cardiovascular disease.	Cohort study
Pavicic-Žeželj et al. [15]	2018	Croatia	Workers in oil and gas companies	*n* = 366	Men: *n* = 177 Women: *n* = 189	37.2 Range <30: 26.8 Range 30–39: 35.1 Range 40–49: 44.0 Range ≥50: 52.3	8.6 Range <30: 1.4 Range 30–39: 2.5 Range 40–49: 2.7 Range ≥50: 1.9	<30 30–39 40–49 ≥50	-	To use the MEDLIFE questionnaire to analyze adherence to the MLS and compare the results with risk factors for cardiovascular pathologies.	Randomized controlled trial
Pérez-Ferre et al. [18]	2015	Spain	Pregnant women with gestational diabetes	*n* = 230 GC: *n* = 111 GI: *n* = 126	Women: *n* = 260	-	-	GC: 32–38 GI: 31–38	-	To perform an intervention in the LS implementing the MLS, in order to prevent glucose alterations in pregnant women with gestational diabetes.	Randomized controlled trial
Sánchez-Villegas et al. [22]	2016	Spain	University graduates	*n* = 11,800	Percentages: MD: T1 Men: 40.1 Women: 59.1 MD: T3 Men: 43.1 Women: 56.9 PA: T1 Men: 33.6 Women: 66.4 PA: T3 Men: 48.1 Women: 51.9 S: T1 Men: 51.9 Women: 48.1 S: T3 Men: 29.2 Women: 70.8	MD: T1: 34.3 MD: T3: 41.3 PA: T1: 36.8 PA: T3: 37.9 S: T1: 42.0 S: T3: 32.4	MD: T1: 10.0 MD: T3: 10.9 PA: T1: 10.9 PA: T3: 12.0 S: T1: 10.8 S: T3: 10.6	-	-	To analyze the relationship between depression and MLS, based on diet (MD), physical activity (PA), and socialization (S).	Cohort study
Sotos-Prieto et al. [14]	2021	Spain	General population	*n* = 11,090 Divided into four groups according to MEDLIFE score, from lowest to highest: Q1: *n* = 3. 042 Q2: *n* = 3. 435 Q3: *n* = 2. 917 Q4: *n* = 1. 696	Men: *n* = 5910 Women: *n* = 5181 Q1: Men: *n* = 1372 Women: *n* = 1670 Q2: Men: *n* = 1614 Women: *n* = 1821 Q3: Men: *n* = 1386 Women: *n* = 1531 Q4: Men: *n* = 807 Women: *n* = 889	Q1: 47.8 Q2: 45.8 Q3: 45.9 Q4: 46.3	Q1: 17.0 Q2: 16.1 Q3: 16.0 Q4: 15.0	-	-	To evaluate the MLS and its relationship with the risk of suffering from cardiovascular diseases.	Cohort study
Sotos-Prieto et al. [5]	2014	Spain	Workers at an automobile assembly plant	*n* = 988	-	-	-	40–55	-	To design a questionnaire that measures MLS adherence.	Cohort study
Sotos-Prieto et al. [35]	2015	Spain	Public school workers and family members involved in the school environment	*n* = 196	Men: *n* = 30 Women: *n* = 166	41.4	9.2	-	-	To study the reliability of the MEDLIFE questionnaire as a research tool.	Randomized controlled trial
Toobert et al. [31]	2005	USA	Post-menopausal women with type 2 diabetes mellitus	*n* = 279 GC: *n* = 116 GI: *n* = 163	Women: *n* = 279	61	-	39–74	-	To intervene in a population sample in which LS changes are implemented based on the MLS.	Randomized controlled trial
Toobert et al. [32]	2010	USA	Post-menopausal women with type 2 diabetes mellitus	*n* = 279 GC: *n* = 116 GI: *n* = 163	Women: *n* = 279	61	-	39–74	-	To examine the long-term effects of healthy behavioral changes following the implementation of the MLS program.	Randomized controlled trial

Lifestyle; MD: Mediterranean Diet; MLS: Mediterranean Lifestyle; Q: quartile; T: Tercile; GC: control group; MDG: group receiving a Mediterranean Diet intervention; MLG: group receiving a Mediterranean Lifestyle intervention; GI: intervention group; MD: T1: variable analysis group Mediterranean Diet: Tercile 1; MD: T3: variable analysis group Mediterranean Diet: Tercile 3; MLS: T1: variable analysis group Mediterranean Lifestyle: Tercile 1; MLS: T3: variable analysis group Mediterranean Lifestyle: Tercile 3; S: T1: variable analysis group socialization: Tercile 1; S: T3: variable analysis group socialization: Tercile 3.

### 3.5. Components of the Mediterranean Lifestyle Evaluated in Each Study

Table 4 includes the MLS components evaluated in each study. To analyze these components, the guidelines published in the study by Diolintzi et al. [2] were followed. The table shows that no article fully evaluated the MLS since none of them included participation in food preparation. MD adherence and the practice of physical activity (PA) were evaluated in all of them. Four studies focused on these two components [10,18,24,33], five also included night-time sleep [19,20,21,23,28], and three included MD, PA, and socialization [18,31,32]. Another study included MD, PA, and the use of locally grown, seasonal products [34]. Only six articles evaluated all of the MLS components, with the exception of the participation in food preparation and the consumption of locally grown and seasonal products [5,11,14,15,35,36]. Moreover, only two articles assessed all but one of the components, food preparation [16,17].

### 3.6. Evaluation Strategies Used to Analyze MLS Components

Table 5 was created with the purpose of organizing all of the extracted information and summarizing the different evaluation strategies used by the authors to analyze each MLS component.

It shows all of the MLS components together with the questionnaires or self-reported ad hoc questions that were used to evaluate each dimension, with the exception of six studies, which exclusively used a tool to analyze the MLS components globally. The MEDLIFE questionnaire, the MedCOVID-19 score, the Total Lifestyle Index (TLI), MEDiLIFE-index, and MEDI-Lifestyle index were used [12,16,23,28,34,36]. It should be noted that, in one of the studies, instead of applying the previously validated 28-item MEDLIFE questionnaire, Bowden et al. [16] used an initial pilot 32-item questionnaire that was created by the original authors, which included 4 questions that could not be validated in a second study [35]. In addition to all the ad hoc questions used for the assessment of each MLS component, both the MEDiLIFE-index and MEDI-Lifestyle indices do not include the assessment of MD adherence, but require a specific questionnaire for their evaluation, and therefore include the KIDMED and PREDIMED questionnaires, respectively. In this sense, the TLI index is made up of different questionnaires that assess each MLS dimension separately, such as the MedDiet Score questionnaire for MD adherence, the Athens Physical Activity Questionnaire (APAQ), the sleep scale of the Medical Outcomes Study (MOS), and the Sleep Index II.

In addition, seven articles used the MEDLIFE tool in combination with other questionnaires to evaluate certain dimensions of the MLS separately [5,11,13,14,15,17,35]. Hershey et al. [17] modified the original MEDLIFE questionnaire, making variations in a total of nine items.

Regarding the other studies, each MLS component was evaluated using different, previously validated, and specific questionnaires [10,18,19,20,21,22,24,25,26,27,31,32].

**Table 5:** Assessment tools used to analyze MLS components.

Author(s)	Mediterranean Lifestyle Components							
	Mediterranean Diet	Hydration	Use of Seasonal/Locally Grown Products	Participation in Food Preparation	Physical Activity	Socialization	Rest (Naps)	Sleep (h/Night)
Anastasiou et al. [23]	MedDiet Score [37]	-	-	-	Athens Physical Activity Questionnaire (APAQ) [38]	-	-	Medical Outcomes Study (MOS) Sleep Scale [39] Sleep Index II [40]
Baldini et al. [24]	Food Frequency Questionnaire (FFQ/FFQ-143/FFQ-136/FFQ-76) [41] MedDiet Score [37]	-	-	-	International Physical Activity Questionnaire (IPAQ) [42]	-	-	-
Bonaccio et al. [34]	MedCOVID-19 [34]	-	MedCOVID-19 [34]	-	MedCOVID-19 [34]	-	-	-
Bouzas et al. [10]	Food Frequency Questionnaire (FFQ/FFQ-143/FFQ-136/FFQ-76) [41] MedDiet Score [37]	-	-	-	Nurses’ Health Study [43] Minnesota-REGICOR [44,45]	-	-	-
Bowden et al. [16]	MEDLIFE [35]	MEDLIFE [35]	MEDLIFE [35]	-	MEDLIFE [35]	MEDLIFE [35]	MEDLIFE [35]	MEDLIFE [35]
Georgousopoulou et al. [25]	Food Frequency Questionnaire (FFQ/FFQ-143/FFQ-136/FFQ-76) [41] MedDiet Score [37]	-	-	-	International Physical Activity Questionnaire (IPAQ) [42]	Short ad hoc self-reported questions	Ad hoc dichotomous questions	-
Georgoulis et al. [19]	Food Frequency Questionnaire (FFQ/FFQ-143/FFQ-136/FFQ-76) [41] MedDiet Score [37]	-	-	-	International Physical Activity Questionnaire (IPAQ) [42]	-	-	Short ad hoc self-reported questions
Georgoulis et al. [20]	Food Frequency Questionnaire (FFQ/FFQ-143/FFQ-136/FFQ-76) [41] MedDiet Score [37]	-	-	-	International Physical Activity Questionnaire (IPAQ) [42]	-	-	Short ad hoc self-reported questions
Georgoulis et al. [21]	Food Frequency Questionnaire (FFQ/FFQ-143/FFQ-136/FFQ-76) [41] MedDiet Score	-	-	-	International Physical Activity Questionnaire (IPAQ) [42]	-	-	Short ad hoc self-reported questions
Grosso et al. [26]	Food Frequency Questionnaire (FFQ/FFQ-143/FFQ-136/FFQ-76) [41]	-	-	-	International Physical Activity Questionnaire (IPAQ) [42]	Short ad hoc self-reported question	-	Pittsburgh Sleep Quality Index (PAQI) [46]
Hershey et al. [11]	Food Frequency Questionnaire (FFQ/FFQ-143/FFQ-136/FFQ-76) [41] MEDLIFE [35]	MEDLIFE [35]	-	-	MEDLIFE [35]	MEDLIFE [35]	MEDLIFE [35]	MEDLIFE [35]
Hershey et al. [17]	Food Frequency Questionnaire (FFQ/FFQ-143/FFQ-136/FFQ-76) [41] MEDLIFE [35]	MEDLIFE [35]	MEDLIFE [35]	-	MEDLIFE [35]	MEDLIFE [35]	MEDLIFE [35]	MEDLIFE [35]
Hershey et al. [36]	FFQ-136 [41] MEDLIFE [35]	MEDLIFE [35]	MEDLIFE [35]		MEDLIFE [35] Nurses’ Health Study [43]	MEDLIFE [35]	MEDLIFE [35]	MEDLIFE [35]
Katsagoni et al. [28]	Food Frequency Questionnaire (FFQ/FFQ-143/FFQ-136/FFQ-76) [41] MedDiet Score [37]	-	-	-	Athens Physical Activity Questionnaire (APAQ) [38]	-	Ad hoc dichotomous question	Short ad hoc self-reported question Athens Insomnia Scale [47]
Katsagoni et al. [27]	KIDMED (included in the MEDiLIFE-index questionnaire) [48]	-	-	-	Short ad hoc self-reported question	-	-	Short ad hoc self-reported question
Lan et al. [12]	PREDIMED [49]	-	-	-	Short self-reported questions (h/week), based on the Metabolic Equivalent Activity Index (MET)	-	Ad hoc dichotomous question	Ad hoc dichotomous question
Marventano et al. [33]	Food Frequency Questionnaire (FFQ/FFQ-143/FFQ-136/FFQ-76) [41]	-	-	-	International Physical Activity Questionnaire (IPAQ) [42]	-	-	-
Mata-Fernández et al. [13]	Food Frequency Questionnaire (FFQ/FFQ-143/FFQ-136/FFQ-76) [41] MEDLIFE [35]	MEDLIFE [35]	-	-	MEDLIFE [35]	MEDLIFE [35]	MEDLIFE [35]	MEDLIFE [35]
Pavicic-Žeželj et al. [15]	Food Frequency Questionnaire (FFQ/FFQ-143/FFQ-136/FFQ-76) [41] MEDLIFE [35]	MEDLIFE [35]	-	-	International Physical Activity Questionnaire (IPAQ) [42] MEDLIFE [35]	Short ad hoc self-reported question MEDLIFE [35]	Short ad hoc self-reported question MEDLIFE [35]	Short ad hoc self-reported question MEDLIFE [35]
Pérez-Ferre et al. [18]	Food Frequency Questionnaire (FFQ/FFQ-143/FFQ-136/FFQ-76) [41]	-	-	-	Three questions taken from the “Lifestyle questionnaire” [50]	-	-	-
Sánchez-Villegas et al. [22]	Food Frequency Questionnaire (FFQ/FFQ-143/FFQ-136/FFQ-76) [41]	-	-	-	Short self-reported questionnaire of a total of 17 activities (h/week), based on the Metabolic Equivalent Activity Index (MET)	Short ad hoc self-reported question	-	-
Sotos-Prieto et al. [14]	Food Frequency Questionnaire (FFQ/FFQ-143/FFQ-136/FFQ-76) [41] MEDLIFE [35]	MEDLIFE [35]	-	-	Nurses’ Health Study [43] Health Professionals Follow-up Study (HPFS) physical activity questionnaires [51,52] MEDLIFE [35]	Short ad hoc self-reported question MEDLIFE [35]	Short ad hoc self-reported question MEDLIFE [35]	Short ad hoc self-reported question MEDLIFE [35]
Sotos-Prieto et al. [5]	Food Frequency Questionnaire (FFQ/FFQ-143/FFQ-136/FFQ-76) [41] MEDLIFE [35]	MEDLIFE [35]	MEDLIFE [35]	-	Nurses’ Health Study [43] Health Professionals Follow-up Study (HPFS) physical activity questionnaires [51,52] MEDLIFE [35]	Short ad hoc self-reported question MEDLIFE [35]	MEDLIFE [35]	Short ad hoc self-reported question MEDLIFE [35]
Sotos-Prieto et al. [35]	MEDLIFE [35]	MEDLIFE [35]	-	-	Validated European Prospective EPIC Cohort Questionnaire [53] MEDLIFE [35]	MEDLIFE [35]	MEDLIFE [35]	MEDLIFE [35]
Toobert et al. [31]	Food Frequency Questionnaire (FFQ/FFQ-143/FFQ-136/FFQ-76) [41]	-	-	-	CHAMBS [54]	UCLA Social Support Inventory [55]	-	-
Toobert et al. [32]	Food Frequency Questionnaire (FFQ/FFQ-143/FFQ-136/FFQ-76) [41]	-	-	-	CHAMBS [54]	Short ad hoc self-reported question UCLA Social Support Inventory [55]	-	-

### 3.7. Indices for the Assessment of MLS

**MEDiLIFE-index** [28]

This index relies on a 3-point scoring system (0-1-2) and has a maximum final score of 8 points (the sum of all questionnaire components). The higher the score, the better the adherence to the MLS.

To evaluate MD adherence, the KIDMED questionnaire was applied. KIDMED ≥ 8 (high adherence) received 2 points; KIDMED 4 to 7 received 1 point; and KIDMED ≤3 received 0 points (weak adhesion). PA was measured as follows: 2 points for PA ≥ 60 min/day; 1 point for PA ≥ 30 and < 60 min/day; and 0 points for PA <30 min/day. For a sedentary lifestyle, 2 points were given for <1 h/d watching TV, videos, screens, etc.; 1 point for ≥1 and ≤2 h/d of sedentary activities; and 0 points for >2 h/d. For sleep, the American Academy of Sleep Medicine Guidelines were followed, taking into account the different age ranges (6–12 years 9/12 h; 13–18years 8/10 h): 2 points were given if the optimal duration was achieved; 1 point if the duration was longer; and 0 points if the duration was shorter. As observed, this questionnaire used short questions, and another questionnaire was used to measure MD adherence (the KIDMED questionnaire).

**MEDI-Lifestyle** [12]

This index consists of seven short ad hoc dichotomous questions. Scores range from 0 to 7, with a score of 7 representing the best degree of adherence, and 0 the poorest. The individual’s weight was evaluated via BMI, receiving 1 point for a BMI <30 kg/m^2^ and 0 points for a BMI ≥ 30 kg/m^2^. Tobacco consumption was also included: 1 point if the person had not smoked in the last 6 months, 0 points if they smoked. The PREDIMED questionnaire evaluated the MD, receiving 1 point for high MD adherence (≥9) and 0 points for poor adherence (≤9). PA was also evaluated, with physically active individuals (≥16 h/week) receiving 1 point and physically inactive individuals (<16 h/week) receiving 0 points. Time watching TV received 1 point for <2 h/d and 0 points for ≥ 2 h/d. For sleep, 1 point was given for 7–8 h/d and 0 points for sleep <7 h or >8 h/d. Regarding naps, 1 point was given if a nap was taken, and 0 points were given if no nap was taken. In this case, two factors were included in the index that are not included in the MLS: weight and smoking.

**Total Lifestyle Index** (TLI) [23]

This index evaluates four dimensions of LS and includes a specific questionnaire for each: diet (MedDiet Score), physical activity (APAQ), sleep quality (MOS and Sleep Index II), and Instrumental Activities of Daily Living (IADL). The results obtained in each questionnaire were divided into quartiles. Values were assigned from 0, for the first quartile (worst score), to 1, 2, and 3 for the other quartiles (higher scores). The total TLI score ranged from 0 to 12. Higher values indicated a more beneficial LS. As in the MEDI-Lifestyle index, a factor that was not part of the MLS was also included: the Instrumental Activities of Daily Living.

**MedCOVID-19 Score** [34]

This questionnaire assesses the current intake of nine foods from the Mediterranean Diet and five MLS-related behaviors, in terms of decreased, maintained, or increased intake, making comparisons between 2019 and autumn 2020.

To estimate the dietary rating, the following scores were assigned:

- Score +1 point for the increased intake of foods that should be consumed more frequently (i.e., fruits, vegetables, legumes, cereals, fish, and olive oil), −1 point if their intake decreased, and 0 points if it remained the same.
- Score +1 point for the lowest self-reported intake of foods that should be consumed less frequently (i.e., meats, dairy products), −1 point for the highest intake, and 0 points if it remained the same.
- Score -1 point if the consumption of alcoholic beverages increased, +1 point if it decreased, and 0 points if it remained the same.
- Score +1 point for all dietary changes in behaviors related to the Mediterranean Lifestyle, i.e., (a) increased consumption of local and (b) ecological food; (c) increased physical activity; (d) decreased intake of home delivery food; and (e) decreased consumption of pre-cooked foods: −1 point for changes in undesired behaviors, and 0 points if they remained the same.

Diet and behavior scores were equaled to obtain a total mark ranging from −14 to 14. Once the score had been calculated, the population was classified as follows: stable population (score = 0), population with an improved MLS (>1), and population with a worsened MLS (score < 0).

**MEDLIFE** questionnaire [5,35]

This questionnaire was designed specifically to evaluate the MLS, without using other supplementary questionnaires to analyze each dimension separately.

It is divided into three blocks. The first block consists of 15 items and measures the consumption of Mediterranean foods. The second block is composed of seven items and measures the habits of the Mediterranean Diet, including hydration. The third block includes six items and measures PA, rest, social habits, and conviviality. The range varies from 0 (low MLS adherence) to 28 (high MLS adherence).

To analyze the reliability of the questionnaire, Cohen’s Kappa coefficient, the intraclass correlation coefficient (ICC), and the limit of agreement (LOA) were used. A comparison was performed using a 142-item questionnaire (full-Q) from which the 28 items constituting the MEDLIFE questionnaire were derived [5,35].

According to the authors, the MEDLIFE questionnaire is a valid instrument to measure MLS adherence in middle-aged adults and can be used for clinical and epidemiological studies in this population. Its generalizability and predictive validity have yet to be examined [35].

In a study by Sotos-Prieto et al. [35], 4 additional questions were included to evaluate food seasonality and moderation, resulting in a 32-item instrument. Despite this, they were not included in the final questionnaire since a comparison with other tools was not possible. Thus, their validity could not be evaluated, and they were excluded from this questionnaire. However, this 32-item questionnaire was used in a recent study by Bowden et al. [16].

The original MEDLIFE questionnaire was also modified in another study. The following specific changes were made [17].

- Block 1 (Mediterranean food consumption):

1. The item relating to the consumption of processed meats was eliminated.
2. The item relating to the consumption of nuts and olives was changed to the consumption of nuts.
3. The item relating to the consumption of herbs, spices, and garnishes was eliminated.

- Block 2 (Mediterranean dietary habits):

1. The item relating to the consumption of wine was changed to the consumption of wine or other common alcoholic beverages.
2. The item “limit nibbling between meals” was removed.
3. The item “consumption of local, seasonal, or organic products” was added.

- Block 3 (PA, rest, social habits, and conviviality):

1. The items “going out with friends” and “practice team sports” were eliminated.
2. The item “time spent eating” was added.

For the modified MEDLIFE questionnaire [17], scores varied from 0 to 26, following the same criteria as the original questionnaire. Although it is indicated that the modified MEDLIFE questionnaire presents a total of 26 items, it only includes 25.

### 3.8. Statistical Analysis Conducted to Create a Mediterranean Lifestyle Score in the Studies Included in the Review

Table 6 shows the different methods that researchers have suggested so far to create a questionnaire or index for assessing the MLS as a global dimension. Thus, most of the studies have employed different statistical analyses to integrate punctuations from diet adherence or dietary intake, sleep quality, or physical activity in a global MLS score. Moreover, the methodologies used to examine the structure of these questionnaires or indices are related to PCA, KMO, and also reliability and validity analyses [5,28,34,35].

## 4. Discussion

This systematic review focused on 26 studies that addressed the different means of assessing the Mediterranean Lifestyle [5,10,11,12,13,14,15,16,17,18,19,20,21,22,23,24,25,26,27,28,31,32,33,34,35,36]. This is the first review of its kind that identifies and analyzes the strategies used in the scientific literature to examine this lifestyle.

MLS is characterized by an adherence to the MD, proper hydration, the use of locally grown and seasonal products, participation in culinary activities, physical activity, and socialization, as well as adequate rest, both at night and through daytime napping [2]. Although numerous studies have supposedly analyzed the MLS, many of them have failed to examine its components as a general construct of lifestyle [10,19,20,21,23,24,25,26,27,28].

All of the reviewed articles assessed the adherence to the MLS components of MD and the practice of PA. The other most frequently evaluated components include socialization, sleep, and napping. The least commonly evaluated MLS dimensions were hydration and the use of seasonal/locally grown products, which were included in the MEDLIFE questionnaire. Moreover, not all of the components making up the MLS were fully evaluated, since none of the studies assessed participation in culinary activities.

Only six articles exclusively used specific strategies to evaluate the MLS [12,16,23,28,34,36], generating the MEDiLIFE-index, MEDI-Lifestyle, Total Lifestyle Index (TLI), MedCOVID-19 Score, and MEDLIFE questionnaires.

Despite the existence of three indices and two specific questionnaires for the evaluation of the MLS, the MEDLIFE questionnaire appears to be the only tool having adequate psychometric properties. However, it has only been validated for an adult population and not for young or elderly populations. While the studies included in this review mainly used validated tools to assess different MLS components, they did so in an independent manner. Only four of the studies relied on global indices that were created through statistical processes to integrate the different evaluated dimensions and perform a global analysis of the MLS [12,23,28,34]. However, after carrying out this systematic review, we have yet to find a tool having these qualities. It has been suggested, therefore, that MEDLIFE is the best instrument for providing an assessment of adherence to the MLS. Until a new tool is developed, this questionnaire appears to be the most appropriate one for analyzing adherence, even though it does not include all the components of this lifestyle [2,5]. Once a new tool with appropriate psychometric properties has been created, it should be validated for the general and clinical population.

Data on the psychometric properties of MLS questionnaires are almost nonexistent. Measures of reliability and validity are quite scarce. Sotos-Prieto et al. [35] and Bonaccio et al. [34] have offered some evidence regarding psychometric properties. The former reported intraclass correlation coefficients and Kappa coefficients that demonstrate the reliability and validity of the MEDLIFE questionnaire, respectively. Bonaccio et al. [34] revealed adequate reliability for the MedCOVID-19 score with a Cronbach’s alpha coefficient of 0.83. However, both internal consistency (via Cronbach’s alpha coefficient) and test–retest reliability and equivalence reliability must be considered in order to reveal the instrument’s level of accuracy with regard to the construction of an assessment [56]. Moreover, scientific papers state that convergent, concurrent, predictive, and construct validity are different gold standards to evaluate this psychometric aspect [56]. They provide information on the relationship between new and validated tools that share the same construct. Katsagoni et al. [28] show a principal component analysis and KMO values, in relation to the structure of the instruments. The Confirmatory Factor Analysis (CFA) is a powerful statistical tool for the development of measurement instruments [57]. CFA and its analytic version, the Exploratory Factor Analysis (EFA), play an essential role in measurement model validation in this regard [57].

Concerning the methods used to create questionnaires for evaluating MLS, four studies included in this systematic review have merely described how researchers have attempted to generate MLS assessment tools. One of them showed a PCA and KMO to determine the intern structure of the questionnaire [28]. Moreover, both Bonaccio et al. and Sotos-Prieto et al. [5,34] also run psychometric analysis such as the reliability of the questionnaire they created. However, it seems extremely difficult to find studies which indicate the psychometric properties of the tools they have created to assess the MLS. As far as psychometrics is concerned, Muñiz and Fonseca-Pedrero [58,59] stated that both qualitative and quantitative methods should be included when constructing a new assessment tool. These authors have indeed suggested that reliability, convergent validity, and factor structure are needed to demonstrate the accuracy and veracity of evaluating the construct [58,60]. It goes without saying that the test construction process needs to be explained in detail, considering all the theoretical and metric principles, since these kinds of studies do not appear to be automatic or universal [58,59,61,62]. Following strictly the guidelines for creating assessment instruments written by Muñiz and Fonseca-Pedrero [58,59], the psychometric model used, the type of item response, the application form, and the assessment context should be considered for the construction of a high-quality evaluation tool.

### Strengths and Limitations

This study offers considerable advances in the examination of evaluation strategies used to analyze the MLS in different populations. However, it has certain limitations that should be taken into consideration. For instance, only four relevant databases were searched. Moreover, although a wide variety of keywords were used, some specific words may not have been identified and included in the search strategies. Moreover, the fact that only articles available in full text were evaluated for inclusion in the revision could also limit the search strategy. However, in this case, all the evaluated studies were found in full text and could be fully assessed for inclusion or non-inclusion in the review. Furthermore, although many studies aim to assess the MLS, they appear to only assess MD adherence or to not explicitly state that MLS is evaluated, potentially hindering the identification of the studies that assess the MLS as a whole. Some articles evaluate two or more components of the MLS but do not name them as such, assigning all of the benefits of this LS exclusively to MD adherence. Therefore, many benefits attributed to the MD may also be derived from MLS adherence, although they are not identified as such in the studies.

Despite these limitations, an exhaustive systematic review was carried out in this study, demonstrating that the MLS is being examined in an increasing number of works. The strengths and limitations of each evaluation strategy performed in the different studies were also evaluated. This analysis provides objective and reliable data on the importance of using a tool with adequate psychometric properties that is capable of performing a comprehensive assessment of all of the dimensions that constitute the MLS.

## 5. Conclusions

The MLS is considered to be a healthy lifestyle in which the frequency and quantity of the consumption of certain foods play a key role. It is also a lifestyle that refers to other dimensions rooted in traditional Mediterranean life, as well as the interrelation between parameters such as socialization, physical activity, leisure activities, proper rest, and diet.

This systematic review attempted to consider all of the methods used to evaluate the MLS. A total of four indices specifically designed for assessment were obtained. However, none of the methods evaluated all of the dimensions that constitute the MLS. Although MEDLIFE may be one of the most reliable and integrating questionnaires for the assessment of the MLS, other psychometric properties of this instrument should be analyzed in depth, such as its factorial structure and functioning in both clinical and healthy populations.

A notable limitation of the current evaluation strategies is the heterogeneity of the tools used to evaluate the MLS, since different authors use distinct methods of analysis. No consensus has yet been reached on a single instrument to comprehensively and reliably measure the MLS that has proven and adequate psychometric properties. Therefore, future studies should attempt to design a tool with appropriate psychometric properties for the general population and include all of the MLS dimensions.

This would allow professionals to carry out more accurate analyses of the level of adherence to a healthy lifestyle and would lead to the identification of populations at risk of developing different pathologies. Moreover, this tool would aid in the creation of comprehensive intervention programs that are aimed at improving health by promoting adherence to a healthy lifestyle, such as the MLS.

## Figures and Tables

**Table 1:** Search strategy.

Search Strategy
1. Mediterranean AND lifestyle (Title/Abstract/keyword)
2. “Mediterranean lifestyle” (Title/Abstract/keyword)
3.“Mediterranean lifestyle” (Title/Abstract/keyword) AND Questionnaire (Title/Abstract/keyword)
4.“Mediterranean lifestyle” (Title/Abstract/keyword) AND Review (Title/Abstract/keyword)
5.“Mediterranean lifestyle” (Title/Abstract/keyword) AND Index (Title/Abstract/keyword)
6.“Mediterranean lifestyle” (Title/Abstract/keyword) AND Evaluation (Title/Abstract/keyword)
7.“Mediterranean lifestyle” (Title/Abstract/keyword) AND Assessment (Title/Abstract/keyword)

**Table 2:** Bibliographic search strategies.

	PubMed	Scopus	Web of Science	Science Direct	Total
Mediterranean AND lifestyle (Title/Abstract/keyword)	1654	3536	2240	517	
“Mediterranean lifestyle” (Title/Abstract/keyword)	71	89	82	25	
“Mediterranean lifestyle” (Title/Abstract/keyword) AND Questionnaire (Title/Abstract/keyword)	10	18	10	0	
“Mediterranean lifestyle” (Title/Abstract/keyword) AND Review (Title/Abstract/keyword)	10	17	11	1	
“Mediterranean lifestyle” (Title/Abstract/keyword) AND Index (Title/Abstract/keyword)	24	27	22	8	
“Mediterranean lifestyle” (Title/Abstract/keyword) AND Evaluation (Title/Abstract/keyword)	5	8	4	5	
“Mediterranean lifestyle” (Title/Abstract/keyword) AND Assessment (Title/Abstract/keyword)	5	22	4	1	
Total	1772	3717	2373	557	8419
Total without duplicates					4707

**Table 4:** Mediterranean Lifestyle components evaluated in each study.

Author(s)	Mediterranean Lifestyle Components							
	Mediterranean Diet	Hydration	Use of Seasonal/Locally Grown Products	Participation in Food Preparation	Physical Activity	Socialization	Rest (Napping)	Sleep (h/Night)
Anastasiou et al. [23]	+	-	-	-	+	-	-	+
Baldini et al. [24]	+	-	-	-	+	-	-	-
Bonaccio et al. [34]	+	-	+	-	+	-	-	-
Bouzas et al. [10]	+	-	-	-	+	-	-	-
Bowden et al. [16]	+	+	+	-	+	+	+	+
Georgousopoulou et al. [25]	+	-	-	-	+	+	+	-
Georgoulis et al. [19]	+	-	-	-	+	-	-	+
Georgoulis et al. [20]	+	-	-	-	+	-	-	+
Georgoulis et al. [21]	+	-	-	-	+	-	-	+
Grosso et al. [26]	+	-	-	-	+	+	-	+
Hershey et al. [11]	+	+	-	-	+	+	+	+
Hershey et al. [17]	+	+	+	-	+	+	+	+
Hershey et al. [36]	+	+	+	-	+	+	+	+
Katsagoni et al. [28]	+	-	-	-	+	-	+	+
Katsagoni et al. [27]	+	-	-	-	+	-	-	+
Lan et al. [12]	+	-	-	-	+	-	+	+
Marventano et al. [33]	+	-	-	-	+	-	-	-
Mata-Fernández et al. [13]	+	+	-	-	+	+	+	+
Pavicic-Žeželj et al. [15]	+	+	-	-	+	+	+	+
Pérez-Ferre et al. [18]	+	-	-	-	+	-	-	-
Sánchez-Villegas et al. [22]	+	-	-	-	+	+	-	-
Sotos-Prieto et al. [14]	+	+	-	-	+	+	+	+
Sotos-Prieto et al. [5]	+	+	-	-	+	+	+	+
Sotos-Prieto et al. [35]	+	+	-	-	+	+	+	+
Toobert et al. [31]	+	-	-	-	+	+	-	-
Toobert et al. [32]	+	-	-	-	+	+	-	-

**Table 6:** Statistical analysis to create a Mediterranean Lifestyle score in the studies included in the review that have created an index or questionnaire.

Authors	MLS Index/Questionnaire	Psychometric Analysis	Global MLS Component
Anastasiou et al. [23]	TLI: Total Lifestyle Index	-	The total score is calculated by adding up the scores of the index’s dimensions distributed into different quartiles. The global MLS score ranges from 0 to 12.
Katsagoni et al. [28]	MEDiLIFE-index.	Principal Component Analysis (PCA) > 0.3 Kaiser–Meyer–Olkin (KMO) = 0.5	The total score is calculated by adding up the scores of the index’s dimensions. Each dimension is evaluated by a 3-point rating scale (0-1-2) The global MLS score ranges from 0 to 8.
Lan et al. [12]	MEDI-Lifestyle	-	The total score is calculated by adding up the scores of the index’s dimensions. Each dimension is categorized dichotomously (0-1). The global MLS score ranges from 0 to 7.
Bonaccio et al. [34]	MedCOVID-19 Score	Reliability: Internal consistency Cronbach’s alpha coefficient = 0.83	The global score is obtained by adding up all the dimensions’ scores. The dimensions are scored from −1 to +1. This total MLS score ranges from −14 to 14.
Sotos-Prieto et al. [5,35]	MEDLIFE	Convergent validity:	The total score is calculated by adding up the scores of all the items. Each item was scored dichotomously (0-1). The total MLS score ranges from 0 to 28.
		− Degree of correlation between the two instruments: 0.626	
		Reliability:	
		− Internal consistency Cronbach’s alpha coefficient = 0.75 − Inter-rater correlation coefficient: 0.544 − Limits of agreement: from 4.66 to 7.45 (Mean = 1.40) − Kappa coefficient: Very good concordance (k = 0.81–1) was observed for ‘limit salt in meals’, ‘nibbling’, and ‘nap’ (10.7% of the items). Good (k = 0.61-0.80) to moderate (k = 0.41–0.60) agreement was found for most of the items evaluated (21.4%) such as wine, moderate consumption of red meat, legumes, fruit, and olive oil consumption) and fair (0.21–0.40) for 32.1% of the items.	

## Data Availability

Data sharing not applicable. No new data were created or analyzed in this study. Data sharing is not applicable to this article.

## Supplementary Materials

The following supporting information can be downloaded at: https://www.mdpi.com/article/10.3390/nu14194179/s1, Table S1. Methodological quality assessment of the cross-sectional studies included in the review; Table S2. Methodological quality assessment of the randomized controlled trials included in the review; and Table S3. Methodological quality assessment of the cohort studies included in the review.
