# Supplementary material for: Assessment Strategies to Evaluate the Mediterranean Lifestyle: A Systematic Review
Source: Nutrients. 2022 Oct 8;14(19):4179. doi: 10.3390/nu14194179 (PMC9571868; doi:10.3390/nu14194179)
Supplement: Supplementary file 1 [file nutrients-14-04179-s001.zip › nutrients-1926046-supplementary.pdf]

Table S1. Methodological quality assessment of the cross-sectional studies included in the review.

Autores	Ítem 1	Ítem 2	Ítem 3	Ítem 4	Ítem 5	Ítem 6	Ítem 7	Ítem 8
Anastasiou et al. (17)	+	+	+	+	+	+	+	+
Baldini et al. (31)	+	+	+	+	-	NA	+	-
Bonaccio et al. (36)	?	+	+	+	+	+	+	+
Bowden et al. (18)	+	+	+	+	+	+	+	+
Georgousopoulou et al. (33)	+	+	+	+	?	-	+	+
Katsagoni et al. (27)	+	+	+	+	+	+	+	+
Lan et al. (28)	+	+	+	+	-	+	+	+
Pavicic-Žeželj et al. (38)	+	+	+	+	-	-	+	+
Sotos-Prieto et al. (5)	-	-	+	+	-	-	+	+

Table S2. Methodological quality assessment of the randomized controlled trials included in the review

Autores	Ítem 1	Ítem 2	Ítem 3	Ítem 4	Ítem 5	Ítem 6	Ítem 7	Ítem 8	Ítem 9	Ítem 10	Ítem 11	Ítem 12	Ítem 13
Bouzas et al. (19)	+	-	+	-	-	?	+	+	+	+	+	+	+
Georgoulis et al. (20)	+	+	+	+	+	?	+	+	+	+	+	+	+
Georgoulis et al. (21)	+	+	+	+	+	-	+	+	+	+	+	+	+
Georgoulis et al. (22)	+	+	-	+	+	+	+	+	+	+	+	+	+
Hershey et al. (28)	+	?	-	?	?	?	?	-	+	+	+	+	?
Katsagoni et al. (29)	+	+	+	+	+	+	+	+	+	+	+	+	+
Pérez-Ferre et al. (23)	+	+	+	?	?	?	+	+	+	+	+	+	?

Sotos-Prieto et al. (39)	+	?	?	?	?	?	?	+	+	+	+	+	+
Toobert et al. (24)	+	?	+	?	?	?	+	+	+	+	?	+	+
Toobert et al. (25)	+	-	+	-	-	-	+	+	+	+	+	+	+

Table S3. Methodological quality assessment of the cohort studies included in the review

Autores	Ítem 1	Ítem 2	Ítem 3	Ítem 4	Ítem 5	Ítem 6	Ítem 7	Ítem 8	Ítem 9	Ítem 10	Ítem 11
Grosso et al. [30]	+	+	+	+	-	?	+	-	+	NA	+
Hershey et al. [34]	+	+	+	+	+	+	+	+	+	+	+
Hershey et al.[38]	+	+	+	+	+	+	+	+	+	NA	+
Marventano et al. [30]	+	+	+	-	-	?	+	-	NA	NA	+
Mata-Fernández et al. [35]	+	+	+	+	+	+	+	+	+	+	+
Sánchez-Villegas et al. [36]	+	+	+	+	+	+	+	+	+	+	+
Sotos-Prieto et al. [37]	+	+	+	+	+	+	+	+	+	+	+
